# Building a sustainable framework for laboratory animal welfare and ethics education: challenges and reform strategies

**DOI:** 10.3389/fvets.2025.1684284

**Published:** 2025-12-18

**Authors:** Jian Zhang, Lingyan Zhang

**Affiliations:** Institute of Laboratory Animal Science, Guizhou University of Traditional Chinese Medicine, Guiyang, China

**Keywords:** curriculum reform, ethical culture, ethics education, institutional governance, laboratory animal welfare

## Abstract

China has established a relatively comprehensive laws and regulations for laboratory animal welfare and ethics (LAWE). In accordance with regulatory requirements, most institutions have set up institutional animal care and use committee (IACUC) to conduct ethical reviews of laboratory animal welfare. The majority of students demonstrate a high level of awareness of LAWE. However, they often lack comprehensive knowledge of the related principles and struggle to apply them in practice. This paper identifies four core challenges for LAWE education: (1) disjointed curricula; (2) undertrained faculty; (3) weak institutional safeguards; and (4) an underdeveloped ethical culture. To address these issues, we propose a comprehensive reform framework across four dimensions: (1) developing a “Required + Integrated + Tiered” curriculum system that combines theory with practice; (2) establishing a dual-certification mechanism and continuous training for faculty to ensure both ethical and technical competence; (3) implementing a closed-loop governance model including entry thresholds, robust IACUC review, and ongoing oversight; and (4) fostering a campus-wide ethical ecosystem through rituals, peer support, and public engagement. These reforms aim to embed ethical awareness into the entire educational process, cultivate respect for life, and build a sustainable, high-quality biomedical education system rooted in both scientific rigor and humanistic values.

## 1 Introduction

Laboratory animals are an indispensable foundational resource in the biomedical field, serving as both “living reagents” and “human substitutes” that bridge basic research and clinical applications. They play an irreplaceable role in drug development, disease mechanism studies, vaccine evaluation, toxicity testing, and educational training by providing vital physiological, pathological, and pharmacological models (1). These models directly impact the scientific validity, accuracy, and international recognition of experimental results. Moreover, the welfare of laboratory animals is closely tied not only to data quality and research ethics, but also to a nation’s advancement in civilization and the rule of law (2). Therefore, strengthening education on laboratory animal welfare and ethics (LAWE), implementing the “3Rs” principle (Replacement, Reduction, Refinement), and promoting the use of alternative methods have become critical for achieving a harmonious balance between scientific advancement and ethical responsibility, thereby promoting the sustainable development of the life sciences.

Animal welfare during experiments is not only a cornerstone of research ethics but also a fundamental prerequisite for ensuring the scientific validity, reliability, and reproducibility of experimental results. Good animal welfare practices can significantly reduce stress responses, improve the physiological and psychological well-being of laboratory animals, and thus yield more accurate and consistent data—ultimately advancing progress in medicine and life sciences (3). As stand-ins for human health, laboratory animals reflect the moral and ethical standards of a society; their welfare is a direct measure of its level of civilization. Strengthening education on LAWE fosters in medical students a deep respect for life and a sense of ethical responsibility, helping to instill the compassionate values at the heart of the medical profession and encouraging harmony between humans and nature. Therefore, LAWE is not only critical to research quality but also a vital reflection of scientific integrity, and humanistic values.

Using legislation to clarify the requirements for the welfare and ethical management of laboratory animals is an important method of protection in China. In 1988, China issued the “Regulations on the Administration of Laboratory Animals,” stipulating that “personnel engaged in laboratory animal work must care for the laboratory animals and are forbidden from teasing or abusing them.” In 2006, China’s Ministry of Science and Technology (MOST) issued the “Guiding Opinions on the Humane Treatment of Laboratory Animals.” This is the first document at the national level to define requirements for laboratory animal welfare and ethics. It requires institutions that produce or use laboratory animals to set up an Institutional Animal Care and Use Committee (IACUC). The use of laboratory animals should be approved by IACUC. According to the Chinese national standard “Guide for the Ethical Review of Laboratory Animal Welfare” (GB/T 35892-2018), the items for supervision include: personnel qualifications, facility conditions, laboratory animal veterinarians, animal source, animal transport, animal husbandry, animal use, technical procedures, and occupational health and safety. Furthermore, standards such as the Guidelines for the Euthanasia of Laboratory Animals (GB/T 39760-2021), the Working Standards for Laboratory Animal Welfare and Ethics (DB32/T 2911-2016), and the Technical Specifications for the Ethical Review of Laboratory Animal Welfare (DB11/T 1734-2020) have also set out regulations for various aspects of the management of LAWE review (4–6).

Currently, LAWE education at some Chinese universities is characterized by a pattern of “high awareness but weak knowledge, limited practice, and insufficient institutional support.” On the one hand, students generally demonstrate a strong recognition of LAWE, widely acknowledging the importance of the “3Rs” principle and humane treatment of animals (7, 8). On the other hand, their grasp of systematic knowledge is limited. The curriculum lacks depth and breadth, with most students encountering animal welfare content only incidentally during laboratory sessions, rather than through dedicated and structured ethical coursework. Moreover, a clear gap exists between knowledge and practice. While students may understand ethical principles in theory, they often fail to apply them appropriately in practice due to insufficient technical skills, lack of institutional frame-works, or inadequate supervision (9). At the institutional level, deficiencies in ethical review procedures and limited promotion of ethical culture contribute to a vague understanding of regulations among both students and faculty, making it difficult to effectively implement ethical standards (10). Overall, the advancement of laboratory animal welfare education urgently requires a coordinated effort across multiple dimensions, including curriculum development, faculty training, institutional safeguards, and the cultivation of a strong ethical culture.

## 2 Challenges in laboratory animal welfare and ethics education

According to the requirement of “Guiding Opinions on the Humane Treatment of Laboratory Animals” issued by China’s MOST in 2006, most universities in China have established IACUC and implemented formal review systems for LAWE. Consequently, students have developed a strong awareness of LAWE. However, there is a significant gap in students’ knowledge of LAWE, which often fails to keep pace with their scientific training. A survey by Rao et al. (11) of medical students at a university in Dalian showed that 49.23% were completely unaware of the “3Rs” principle and related concepts. In a survey of veterinary medicine students at a university in Beijing, Li et al. (12) found that 25.93% of students did not know the “3Rs” principle or animal welfare, while only 19.26% reported a thorough understanding. Furthermore, a survey by Zou et al. (13) of postgraduates in Beijing revealed that only 34.62% had a detailed knowledge of these principles. Inadequacies in LAWE education have led to a poor acquirement of the subject among a considerable number of students. We believe that LAWE education in China currently faces the following four challenges: (1) disjointed curricula; (2) undertrained faculty; (3) weak institutional safeguards; and (4) an underdeveloped ethical culture.

### 2.1 Curriculum development issues: fragmented content, disjointed structure, and lack of systematic planning

At present, the construction of laboratory animal welfare and ethics curricula in Chinese medical universities is commonly afflicted by three core problems: fragmented content, disjointed structure, and lack of systematic planning. First, the content is severely fragmented. In most universities, LAWE is not taught as a standalone course, but rather embedded in a piecemeal fashion within experimental courses, or merely mentioned in passing by laboratory instructors (9, 14). A survey by Luo et al. (15) showed that students have limited ways for acquiring knowledge of LAWE, with only 53.70% obtaining it through courses and 11.64% through research practice and online sources. The survey by Liu et al. (16) reveals a significant gap between the need for and availability of LAWE education. Although 92.5% of respondents expressed a strong desire for their institutions to implement LAWE training and enhance awareness, a mere 8.8% confirmed having ever attended a relevant course, indicating a severe lack of coverage. While some universities offer elective courses in Laboratory Animal Science, the topic of laboratory animal welfare and ethics is often confined to a single chapter, lacking a systematic coverage. This makes it difficult for students to develop a comprehensive and coherent understanding of ethical principles.

Second, the curriculum structure lacks both hierarchy and continuity. Requiring students to conduct complex animal experiments without a firm grasp of the principles of laboratory animal welfare and ethics compromises animal welfare.

Third, course content is outdated. Most textbooks still center around the “3Rs” principle and fail to incorporate newer frameworks like the “4Rs” or emerging concepts such as Refusal and Responsibility (17). Additionally, they lack up-dated case studies aligned with China’s most recent regulations, such as the Guidelines for Ethical Review of Laboratory Animal Welfare (GB/T 35892-2018).

Moreover, current curricula often prioritize theoretical knowledge over practical competence. Students may be able to recite ethical codes but struggle to apply them in real animal studies (18). A survey of 288 medical graduate students showed that, despite having taken the compulsory course “Medical Laboratory Animal Science,” there were still gaps in both theoretical knowledge and practical experience concerning the LAWE (7).

A more pressing issue is the weak course development capacity in some universities, where “copy-paste” course models prevail. These courses lack integration with actual situation of the university and the major characteristics, resulting in failure to foster genuine ethical awareness and behavior for laboratory animals.

### 2.2 Faculty training issues: lack of specialization, fragmented training, and ineffective incentives

Faculty play a crucial role in delivering LAWE education, yet the current training system faces three major challenges: lack of specialization, fragmented training, and ineffective incentives.

First, there is a serious lack of specialized knowledge. Most laboratory instructors or technicians lack a systematic knowledge of the principles, regulations, and standards for laboratory animal welfare and ethics. Their teaching is often limited to operational norms, without the depth needed to cultivate students’ critical thinking or moral reasoning (19).

Second, faculty training programs are fragmented and overly simplistic. Training is often limited to one-off pre-service workshops or perfunctory continuing education hours, with little long-term planning. The training content typically emphasizes technical procedures over ethical education and theoretical knowledge over practical, context-specific case analysis. It also fails to differentiate instruction based on the instructor’s discipline, the student’s academic level, or the types of experiment involved.

Third, the incentive system is weak. Ethical teaching competence is rarely considered in performance evaluations, promotion, or teaching awards, leaving faculty with little motivation to invest in LAWE education. Some even regard it as an extra burden, delivering it in a perfunctory, box-checking manner.

This problem is even more pronounced in local or provincial medical schools, which often lack access to high-quality training resources. As a result, instructors resort to cobbling together materials from online downloads or borrowed slides from peer institutions, further widening the gap in education quality across regions and institutions.

### 2.3 Institutional safeguard issues: policy gaps, tokenistic review processes, and lack of oversight

Institutional safeguards are the essential framework for implementing LAWE education. However, current practices show systemic failures in the form of missing policies, formalized review procedures, and a vacuum of supervision.

First, there is the issue of policy gaps. While national documents such as the “Regulations on the Administration of Laboratory Animals” and the “Guiding Opinions on the Humane Treatment of Laboratory Animals” have been issued in China, many remain aspirational rather than binding (20). The absence of enforceable implementation measures results in inconsistent practices across regions and institutions. According to incomplete statistics, 30% of the more than 2,000 laboratory animal institutions in China have not established IACUC (21). A survey by Liu et al. (17) and Liu et al. (21) of students at a hospital in Tianjin showed that while more and more graduate students engaged in basic research are becoming familiar with LAWE knowledge, over 90% have not obtained a training certificate, leaving laboratory animal ethics almost entirely at the theoretical level (21). Second, ethical review procedures are highly formalistic. Although many universities have established IACUCs, the review process often emphasizes document compliance over substantive ethical analysis. Reviews focus on whether the protocol appears to meet standards, rather than evaluating ethical risks and pain management in practice (21, 22). In some cases, protocols are submitted after the experiment has already been conducted, undermining the purpose of ethical review (23).

Third, there is a lack of ongoing oversight. Most institutions lack regular ethical inspections and mechanisms for tracking or penalizing violations. Student misconduct—such as performing surgeries without anesthesia or conducting repetitive, unnecessary experiments—often goes unnoticed and unpunished. As a result, regulations may be prominently displayed on walls, but actual behavior remains unchanged. There is a lack of supervision over the procedures during animal experiments (21).

Even more concerning, some institutions perceive LAWE review as a “barrier” to scientific productivity, encouraging faculty to bypass or oversimplify procedures to expedite research outcomes. What’s even more concerning is that some researchers apply for “ethical approval” just before their research results are about to be published, which severely violates research integrity. This attitude further erodes the authority of the ethical system (21). Additionally, the lack of a unified national ethics review information system and a “blacklist” mechanism allow problematic researchers and projects to move between institutions unchecked, creating regulatory loopholes.

### 2.4 Culture development issues: lack of cultural integration, absence of rituals, and emotional detachment

The ultimate goal of LAWE education is the integration of knowledge and action. However, in terms of cultural development, current efforts suffer from a triple disconnection: cultural absence, lack of ritual, and emotional detachment.

First, there is a significant cultural gap on campuses. Most medical schools lack systematic cultural expressions of LAWE. According to a survey by Liu et al. (24) conducted at Chongqing University, only 7.14% (5/70) of the students and 20.00% (4/20) of the teachers were aware of the relevant laws and standards concerning LAWE.

There are few, if any, visible signs—such as ethical posters, memorial spaces, or thematic exhibitions—leaving students in an emotionally neutral environment that fails to foster ethical awareness.

Second, there is a lack of ritualized education. Before experiments, there are typically no ceremonies of respect or moments of silence to evoke reflection. Afterward, animal remains are often discarded carelessly, sometimes even treated as “biological waste,” depriving students of the opportunity to confront the moral weight of taking life.

Third, there is growing emotional detachment. Current education overemphasizes technical rationality, reducing animals to mere research tools and neglecting their value as sentient beings with emotional and moral significance. This has led to a disconnect between knowledge and behavior: students may verbally acknowledge ethical principles but act with cold indifference or even brutality during experiments.

More alarmingly, some students, having received no emotional guidance, develop “experiment desensitization syndrome,” becoming numb to animal suffering or even engaging in mocking or violent behavior. This points to a profound failure of ethical education at the emotional level.

To address this, it is essential to establish cultural mechanisms such as LAWE awareness week, laboratory animal memorials, and commemorative ceremonies involving faculty and students. These initiatives can help restore students’ sense of reverence for life and cultivate a deeper ethical responsibility.

## 3 Reform strategies for laboratory animal welfare and ethics education

**Figure 1:**
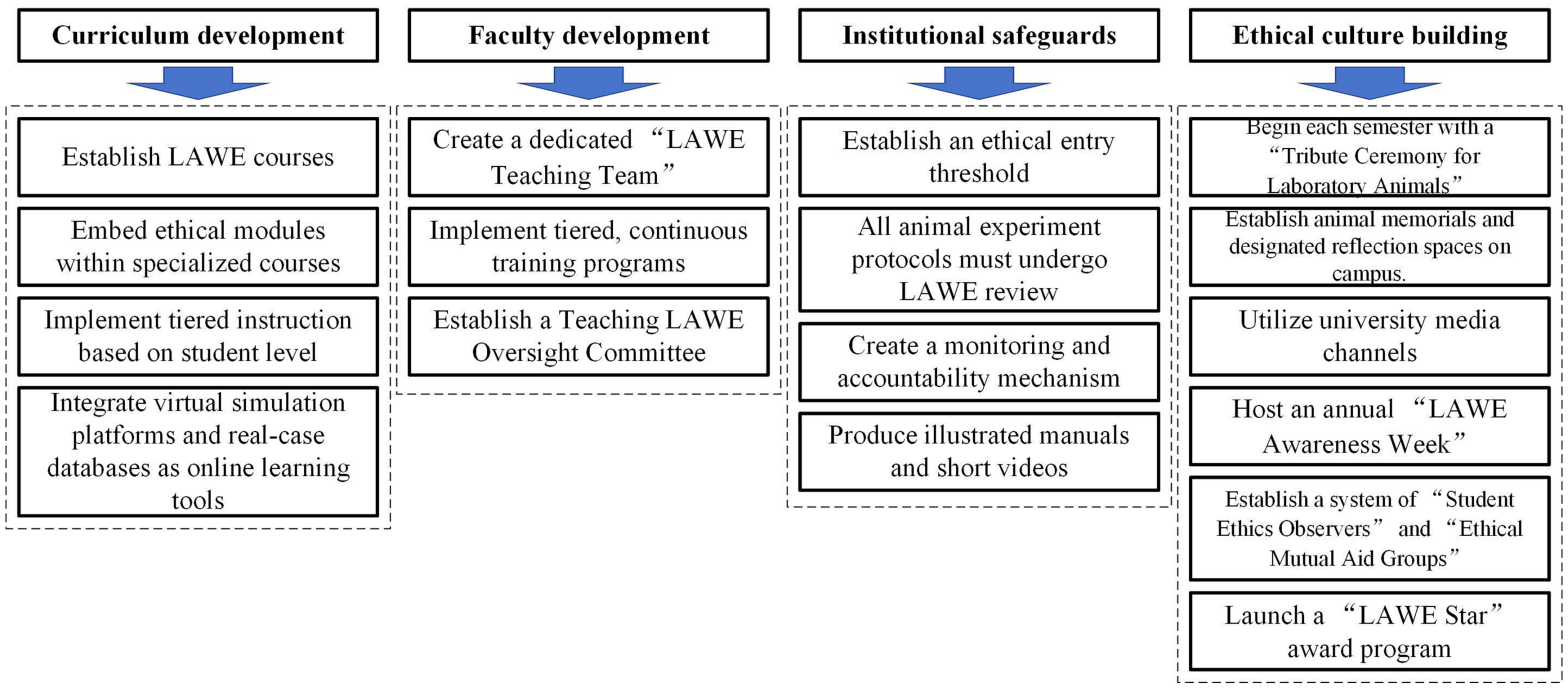
Reform strategies for laboratory animal welfare and ethics education.

To enhance students’ LAWE awareness and knowledge, reforms are suggested to be implemented across four key areas: curriculum development, faculty development, institutional safeguards, and ethical culture building (Figure 1). Universities and institutions can choose appropriate strategies to implement LAWE education based on their specific circumstances.

### 3.1 Curriculum development: from fragmentation to systematization

In many universities, content related to LAWE is still presented in a fragmented manner—scattered across various lab courses or occasional lectures (25). This piecemeal approach leaves students seeing isolated “trees” but missing the ethical “forest,” making it difficult to build a cohesive ethical framework. To cultivate true ethical awareness, a comprehensive “Required + Integrated + Tiered” curriculum system is essential:

First, establish mandatory courses titled “Laboratory Animal Welfare and Ethics” for both undergraduate and graduate students, covering core topics such as the fundamentals of laboratory animal science, the 3Rs/4Rs principles, euthanasia techniques, and domestic and international regulations (26). To provide students with a systematic understanding of laboratory animal-related knowledge, including laws, regulations, standards, LAWE, and basic animal experimentation techniques, our university offers a public elective course in Laboratory Animal Science. To cultivate students’ awareness of LAWE and welfare techniques, our university also offer a public elective course “Laboratory Animal Welfare and Ethics.”

Second, embed ethical modules within specialized courses—such as physiology, surgery, and pharmacology—through activities like simulated ethical reviews and humane endpoint assessments, enabling students to apply ethical standards alongside technical training (10). Wang et al. (19) added LAWE education to the general surgery laboratory course. This way significantly improves students’ awareness of LAWE.

Third, implement tiered instruction based on student level: undergraduates should focus on standardized procedures and emotional development, while graduate students delve into research ethics and the design of alternative methods.

Finally, integrate virtual simulation platforms and real-case databases as online learning tools, and combine them with offline experiential activities like animal memorials and ethical role-playing (27–29). This allows students to experience a progressive and holistic development of knowledge, skills, and emotional awareness within a single course—breaking away from the trap of “learn, test, forget” and moving toward deep learning and retention. A survey from Portugal shows that students have a high acceptance of e-learning in laboratory animal science training (27). Integrating alternative tools with live-animal training enhances technical skill acquisition and supports 3Rs-based laboratory animal practices.

### 3.2 Faculty development: from lack of training to dual certification in ethics and skills

Instructors are the frontline stewards of LAWE education. However, many laboratory instructors or technicians currently lack formal training in ethical theory, and some even struggle to model ethical behavior themselves (30). To address this, institutions should establish a dual-certification system for faculty:

First, create a dedicated “LAWE Teaching Team.” All instructors must undergo formal LAWE training and pass a teaching competency assessment before being approved to teach. Chinese Association for Laboratory Animal Sciences (CALAS) offers level-based training for personnel engaged in laboratory animal science. This training is divided into two categories: laboratory animal technician and laboratory animal veterinarian. Instructors can select the training that best aligns with their professional specialization. Our university is a continuing education base for the CALAS, providing professional training for faculty.

Second, implement tiered, continuous training programs. At the foundational level, offer workshops for all laboratory instructors covering topics such as animal welfare regulations, ethical review procedures, and practical implementation of the 3Rs. At the advanced level, select outstanding faculty to attend professional development programs at international organizations like Association for Assessment and Accreditation of Laboratory Animal Care (AAALAC) or National Centre for the Replacement Refinement & Reduction of Animals in Research (NC3Rs). Upon return, these instructors will lead course development, case study updates, and serve as mentors for junior faculty (31).

Meanwhile, establish a Teaching LAWE Oversight Committee to conduct random audits of ethics-related instruction. Link the quality of LAWE education and student satisfaction to promotion, performance reviews, and teaching awards. Faculty who fails evaluations twice will be temporarily disqualified from teaching lab courses. This creates a closed-loop system of training → assessment → incentives → retraining, ensuring that every instructor is both ethically informed and technically competent.

### 3.3 Institutional safeguards: from voluntary compliance to a closed-loop governance system

LAWE education cannot rely solely on moral appeals; it must be institutionalized to turn “ought to” into “must do.” Universities should implement a comprehensive system covering admission, review, oversight, and accountability:

First, establish an ethical entry threshold: students must complete required ethics coursework and pass a practical exam before receiving laboratory animal personnel certification. No certificate, no access to the lab. Instructors launching experimental courses must also be certified in ethical instruction.

Second, at the institutional level, all animal experiment protocols must undergo LAWE review and include justification for animal use, proposed alternatives, humane endpoints, and euthanasia methods. Approval results should be tied to project funding, graduation qualifications, and thesis defenses. To facilitate LAWE review, a digitalized Animal Ethics Management System (AEMS system) can be used. The AEMS system focuses on various aspects of university animal ethics management, such as the approval process, post-approval monitoring, and project termination, etc. (22).

Third, create a monitoring and accountability mechanism. Establish a LAWE Inspection Team to conduct random checks each semester. Any violations—such as surgery without anesthesia, repetitive unnecessary testing, or animal abuse—should be immediately halted and subject to disciplinary procedures. Violations should be recorded and factored into students’ scholarship and postgraduate recommendation eligibility, as well as faculty evaluation and recognition.

Finally, produce illustrated manuals and short videos outlining all procedures, rules, and expectations. Display these materials prominently in laboratories, animal facilities, and on the university’s homepage. Ensure that ethical knowledge is always visible, accessible, and actionable—turning policy into practice and responsibility into culture.

### 3.4 Ethical culture building: from classroom instruction to a campus-wide ethical ecosystem

LAWE awareness cannot be instilled in a single class; it requires long-term cultural immersion and emotional engagement. Universities should foster an integrated LAWE ecosystem built on ritual, communication, peer support, and recognition:

Begin each semester with a “Tribute Ceremony for Laboratory Animals,” where students and faculty jointly observe a moment of silence and offer flowers—planting the seeds of respect for life from day one.

Establish animal memorials and designated reflection spaces on campus. Regular activities such as flower offerings and message boards can transform these spaces into ethical landmarks. Many universities, including our university, now have laboratory animal monuments and organize commemorative events annually on April 24th, world laboratory animal day.

Utilize university media channels—including official websites, social media, and the Laboratory Animal Center’s homepage—to create a robust “LAWE Corner.” Share updates on laws and regulations, real case studies, and international developments.

Host an annual “LAWE Awareness Week,” featuring poster design contests, student films, public speaking events, and expert panels to keep ethical discourse visible and engaging.

Establish a system of “Student Ethics Observers” and “Ethical Mutual Aid Groups.” Senior students can serve as mentors, helping to review LAWE plans before experiments and evaluate practices afterward, fostering a culture of mutual supervision and growth.

Finally, launch a “LAWE Star” award program. Incorporate LAWE performance into evaluations for outstanding students, graduates, and advisors, making LAWE excellence visible and valued. In doing so, create a positive feed-back loop where “everyone talks ethics, and every act follows standards.”

## 4 The necessity for implementing LAWE education

The implementation of LAWE education is essential for ensuring the ethical use of animals in scientific research and for advancing the overall quality of biomedical studies. We analyze the necessity of implementing LAWE education in universities.

### 4.1 Ensuring ethical standards in research

As scientific research increasingly relies on animal models, it is critical to ensure that these animals are treated humanely and in accordance with ethical guidelines. LAWE education helps researchers understand and apply ethical principles, such as the “3Rs” (Replacement, Reduction, and Refinement), ensuring that animal welfare is prioritized throughout the research process (32). Without proper ethical education, there is a risk of neglecting the welfare of laboratory animals, which could lead to unnecessary harm and invalidate research outcomes. Furthermore, academic journals now require authors to include a statement in the manuscript’s Methods section indicating that the animal research protocol and procedures were ethically reviewed and approved, along with the name of the reviewing body. This underscores the necessity of having well-trained researchers who can meet the ethical standards demanded by international academic journals (33).

### 4.2 Meeting regulatory and ethical compliance requirements

In many countries, including China, regulatory frameworks have been established to govern the use of laboratory animals, such as the “Regulations on the Administration of Laboratory Animals” and the “Guiding Opinions on the Humane Treatment of Laboratory Animals.” Compliance with these regulations is mandatory for obtaining research funding, publishing in academic journals, and gaining institutional approval for research protocols. In China, significant progress has been made in the life sciences and medical fields, including the establishment of a system for laboratory animal welfare and ethics. The publication of the ARRIVE guidelines in 2010 by the UK National Centre for the 3Rs (UKNC3Rs) further reinforced the need for high-quality reporting and transparency in animal research (34). Some journals even require authors to submit an ARRIVE report to ensure compliance with these guidelines. LAWE education is essential to ensure that students, researchers, and faculty members are well-versed in these regulations and can navigate the approval processes, thereby avoiding ethical violations and potential consequences, such as retracted publications or loss of research credibility.

### 4.3 Enhancing research quality and reproducibility

Properly trained researchers who are knowledgeable about animal welfare practices are more likely to produce reliable, reproducible, and scientifically valid results (35). LAWE education contributes to the development of sound research methodologies by reducing the risk of experimental bias, improving the reliability of data, and enhancing the reproducibility of studies. This leads to more robust scientific findings and higher-quality research outcomes. Additionally, awareness of animal welfare standards, such as tumor size limitations for animal models, helps ensure that experiments follow both domestic and international guidelines. For example, according to these guidelines, the tumor diameter in animal models should generally not exceed 20 mm for mice or 40 mm for rats, and no obvious tumor ulceration should be present. This ensures that animal experiments are conducted in a scientifically sound and ethical manner, ultimately improving the reproducibility of the research.

### 4.4 Fostering social responsibility and public trust

As animal research is often scrutinized by the public and advocacy groups, fostering a sense of social responsibility and ethical conduct among researchers is essential. LAWE education instills values of respect for life and ethical responsibility, ensuring that researchers approach their work with integrity and compassion. By training future researchers in LAWE, institutions can build public trust and demonstrate their commitment to ethical standards, ultimately contributing to the social acceptance of animal-based research. The establishment of ethical guidelines, such as those requiring animal protocol approval and ARRIVE reporting, not only promotes transparency but also enhances the integrity of the research process, which is vital for maintaining public confidence in scientific research.

### 4.5 Aligning with international standards

International journals and research organizations increasingly require evidence of ethical review and compliance with established animal welfare standards, such as the ARRIVE guidelines. Implementing LAWE education ensures that researchers are familiar with these international standards and can meet the ethical review requirements for publishing research. This alignment with global standards is crucial for maintaining the reputation and credibility of research institutions and contributing to the advancement of science on a global scale. By adhering to these standards, researchers ensure that their work is not only scientifically rigorous but also ethically responsible, thus supporting the broader goals of scientific integrity and the responsible use of animals in research.

## 5 Conclusion

Although China has established a review system for LAWE, and students exhibit a strong awareness of LAWE, there is a notable lack of theoretical knowledge on the subject. Current LAWE educational practices face significant challenges—including fragmented curricula, under-trained faculty, weak institutional safeguards, and a lack of LAWE culture. Addressing these issues requires a comprehensive, multi-dimensional reform strategy. By systematizing the curriculum, professionalizing faculty development, institutionalizing ethical governance, and cultivating a campus-wide culture of respect for life, universities can transform LAME from a peripheral concern into a core pillar of bio-medical education. It should be noted that the successful implementation of LAWE education reforms requires strong and sustained support from university administration, including commitment to policy development, resource allocation, and integration of LAWE into the broader educational framework. For these reforms to be effective and sustainable, universities must prioritize LAWE education as a vital component of research integrity and ethical responsibility, recognizing its significance in enhancing both the institution’s global reputation and the quality of its scientific output. Ultimately, these efforts will not only improve the quality and reproducibility of scientific research, but also nurture a new generation of ethically grounded biomedical professionals capable of advancing both science and humanity.

## Data Availability

The original contributions presented in the study are included in the article/supplementary material, further inquiries can be directed to the corresponding author.
